# Adaptation and Validation of the Post-Pandemic Health Promotion Behavior of Young Adults in the Digital Age (PS-SGD) Scale in the Turkish Population

**DOI:** 10.3390/healthcare12131337

**Published:** 2024-07-05

**Authors:** Mustafa Can Koç, Elif Yıldırım, Rabia Hurrem Ozdurak Singin, Laurentiu-Gabriel Talaghir, Teodora Mihaela Iconomescu, Neşe Karakaş

**Affiliations:** 1 School of Physical Education and Sports, Istanbul Gelisim University, 34310 Istanbul, Türkiye; 2 Directorate of Sports Sciences Application and Research Center, Istanbul Gelisim University, 34310 Istanbul, Türkiye; 3 Department of Statistics and Quality Coordinator, Konya Technical University, 42250 Konya, Türkiye; edil@ktun.edu.tr; 4 Faculty of Health Sciences, Department of Exercise and Sport Sciences, Malatya Turgut Ozal University, 44000 Malatya, Türkiye; hurremo@gmail.com; 5 Faculty of Physical Education and Sport, Dunarea de Jos University of Galati, 800008 Galati, Romania; ticonomescu@ugal.ro; 6 Faculty of Medicine, Department of Public Health, Malatya Turgut Ozal University, 44000 Malatya, Türkiye; nese.karakas@ozal.edu.tr

**Keywords:** Turkish population, young adults, post-pandemic, health promotion behavior

## Abstract

Background: Young adulthood is a critical developmental period in which individuals establish life-long health behaviors and take responsibility for their own health care. Health promotion strategies tailored to young adults, leveraging digital tools, and addressing challenges exacerbated by events like the COVID-19 pandemic are needed. The aim of this study was to adapt the post-pandemic health promotion behavior of young adults in the digital age (PS-SGD) scale to the Turkish population in order to assess and compare the health behavior of young adults after the pandemic. Methods: A total of 312 participants, aged between 19 and 29 years, were included in the study via non-probabilistic criterion sampling, while the Turkish adaptation process started with translation and back translation methods performed with three language and two health science experts. For statistical analysis, EFA and CFA were conducted to evaluate internal consistency and structural validity. Confirmatory factor analysis was utilized to confirm the structure of the six sub-dimensions. Additionally, measurement invariance was examined regarding participants’ gender to determine if the scale accurately captured similar traits across diverse groups. The relationship between the test–retest data was tested by Pearson correlation to measure consistency and its invariance over time. Results: The gender distribution of the sample was found to be 61.3% female and 38.7% male. According to the results of EFA, items 8 and 18 were removed from the Turkish-adapted version. As a result of the reliability analysis conducted with the Turkish version of the scale, the Cronbach alpha coefficient was obtained as 0.851 for the post-pandemic health promotion behavior. Additionally, the scale was rated as reliable with the following Cronbach alpha values: 0.79 for the “personal hygiene”, 0.78 for “dietary habits”, 0.72 for “using mobile devices”, 0.70 for “emotional health”, 0.68 for “health care and physical activity”, and 0.51 for “social health” sub-dimensions. To examine the six sub-dimension factor structures of the scale, fit indices were calculated as χ^2^/df (1.722), GFI (0.894), IFI (0.908), TLI (0.892), CFI (0.907), RMSEA (0.048), and SRMR (0.057) and were within acceptable limits. Findings of the multi-group confirmatory factor analysis for measurement invariance were less than or equal to 0.01 for the ∆CFI and ∆RMSEA values across all indices. Consequently, it was observed that the item–factor structure, factor loadings, variances, covariances, and error variances of the scale were equivalent for both male and female young adults, while test–retest results showed a high positive correlation. Conclusions: The Turkish version of the post-pandemic health promotion behavior scale of young adults in the digital age scale, consisting of 25 items and six subscales, was proven to be a valid and reliable tool to measure health promotion behavior in young adults aged 19–29 years.

## 1. Introduction

Non-communicable diseases (NCDs) like diabetes, cancer, cardiovascular, and chronic respiratory diseases contribute to approximately 70% of global deaths [1,2]. These diseases are largely influenced by unhealthy behaviors such as physical inactivity, smoking, unhealthy diet, risky sexual behaviors, and alcohol consumption, which are often adopted during youth [3,4]. Young people are prone to experimentation and exploration, leading to a high risk of adopting unhealthy behaviors [5]. Young adulthood is a critical developmental period in which individuals establish life-long health behaviors and take responsibility for their own health care [6]. Most of the health problems of adolescents and young adults are preventable and should be the focus of assessment and intervention [7,8]. Health promotion, which includes education and improving literacy, plays a crucial role in encouraging healthy behaviors and lifestyles from a young age to prevent NCDs [9,10,11,12,13]. The concept of health promotion is defined as “the process that enables people to improve their health and increase their control over the determinants of their health” [14]. Health promotion has been an important strategy in health policies to increase the health status of the whole society. This definition moves beyond a focus on an individual’s behavior to a wide range of potential social and environmental interventions [15]. The accelerated development of technology and digitalization plays a major role in the progress of health promotion. The importance of the Internet in obtaining health information has created a multitude of digital health information resources that help individuals discover ways to improve and maintain personal health [16]. The current increase in electronic health information sources, such as different websites and hundreds of mobile health applications, continues to become increasingly important in accessing health and medical information [17,18]. A study showed that 86.7% of medical school students think that access to health resources on the internet plays an important role during the pandemic, and 65.2% think that the internet is useful when making decisions about their health [19]. However, challenges such as the ability to translate information into behavioral change were highlighted during the COVID-19 pandemic, where lifestyle habits worsened due to factors like increased sedentary behavior, reduced physical activity, and unhealthy dietary changes during lockdowns [20,21]. During that period, health information found online changed rapidly and was inadequate during the pandemic [22].

During the pandemic, young adults were more sedentary and consumed more screen time because of boredom, social isolation, and to stay informed about COVID-19 [23,24]. The lockdown was associated with negative psychological effects, including confusion and anger [25,26], while extended exposure to social media has been associated with a high prevalence of mental health problems [27]. Current studies demonstrate that young adults’ well-being was affected by the COVID-19 pandemic due to the social restrictions [28], and symptoms of anxiety, posttraumatic stress and depression have increased since the onset of the pandemic [29]. The pandemic’s effect on health behavior increased the use of alcohol, daily sitting time, and sedentary behavior [30,31,32]. Home confinement may also negatively impact sleep quality [33,34] and nutritional habits [35,36]. The pandemic also had profound economic, social, and psychological impacts on young adults, including increased stress, sedentary behavior, and negative effects on mental health. Studies across various countries indicated shifts towards less physical activity, increased screen time, and changes in alcohol and tobacco use in Canada [37], Italy [38,39], Brazil [40], and Poland [41]. A systematic review stated that the time spent on exercise decreased from 38.65 to 32.06 min/day and screen time increased from 5.06 to 6.79 h per day during the pandemic compared to pre-pandemic conditions. In contrast, fast food consumption decreased from 37.7% before the pandemic to 33.3% during the pandemic. The percentage of heavy drinkers increased from 20.9% to 25.7%, while tobacco use increased from 5.8% to 7.9% during the pandemic [42]. These findings indicate that there is an urgent need to further assess pandemic-related increases in unhealthy behavior [43,44].

Overall, health promotion strategies tailored to young adults, leveraging digital tools and addressing challenges exacerbated by events like the COVID-19 pandemic are important aspects. The need for effective interventions to promote and sustain healthy behaviors among young populations globally is mostly underscored. The aim of this study was to adapt the post-pandemic health promotion behavior of young adults in the digital age (PS-SGD) scale to the Turkish population in order to assess and compare the health behavior of young adults after the pandemic.

## 2. Materials and Methods

### 2.1. Participants

**Figure 1:**
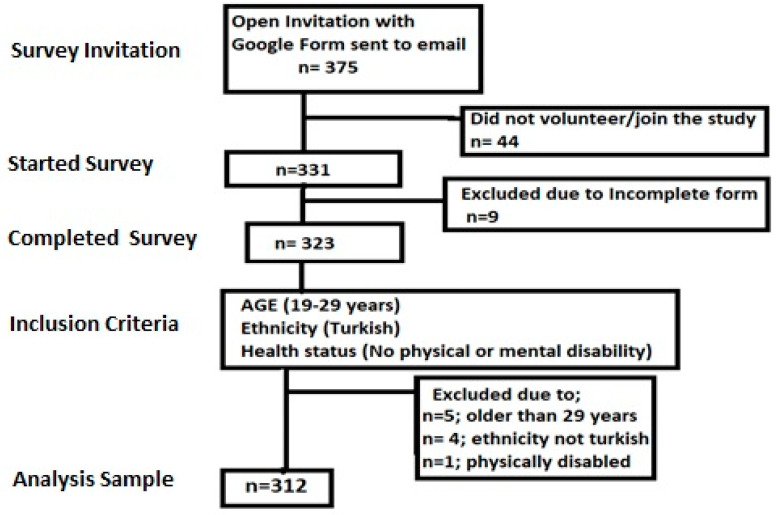
Flowchart of participant recruitment process.

The cross-sectional study was conducted in accordance with the Declaration of Helsinki, with the approval of the Istanbul Gelisim University ethics committee (2 September 2024/February 2024). Although different approaches were used to determine the sample size in studies applying factor analysis, we preferred to use the most accepted sampling procedure, with at least ten participants per item [45,46]. Thus, the sample size was calculated as 270 participants for the Turkish adaptation of the original scale consisting of 27 items. In order to tolerate drop-outs and incomplete sheets, all students of the Recreation Department of Sport Sciences were selected as participants. A total of 375 participants were invited to the study based on the non-probabilistic convenience sampling method by sending the online form of the survey to the student account of each student. A total of 331 responses have been received, while only 312 participants’ data were eligible according to the inclusion criteria. The data of 19 participants were excluded since 5 participants were older than 29 years, 9 participants had missing information and/or incomplete survey form, 4 participants were foreign students with a different ethnicity, and 1 participant was disabled. The flow chart of the participant recruitment process is given in Figure 1.

### 2.2. Instruments and Procedure

The original PP-HPB scale developed by Heo et al. [47] consists of 27 items grouped in 6 sub-dimensions; emotional and social health (ESH) (item 1–8), personal hygiene (PH) (item 9–13), dietary habits (DH) (item 14–18), health care (HC) (item 19–20), using mobile devices (UMD) (item 21–24), and physical activity (PA) (item 25–27). The 5-point Likert scale is rated ranging from ‘almost always (5)’ to ‘never (1)’ for each item, while each score has been summed-up and divided by the item number to find the score for each sub-dimension. The validity and reliability study of the original scale was conducted on 446 young adults and Cronbach alpha value of the scale was calculated as 0.90, while Cronbach alpha values of subscales were calculated as 0.86 for ESH sub-dimension, 0.80 for PH sub-dimension, 0.81 for DH sub-dimension, 0.57 for HC sub-dimension, 0.73 for UMD sub-dimension, and 0.72 for PA sub-dimension.

In order to determine the post-pandemic health promotion behaviors of young adults aged 19–29 years in the Turkish society, the (PP-HPB) scale was adapted to Turkish as described below.

After receiving the permission of the scales’ owner via email, the adaptation process started with the translation of the scale to Turkish language. Translation and back-translation method was used for language adaptation such that all items in the original English form were translated into Turkish by three linguists who are competent in English grammar. As the second step, the Turkish forms were translated from the target language back to the original language by a linguist experienced in scale adaptation, and the consistency between the original English form and the Turkish form was established. Finally, to investigate the language and content validity of the final Turkish form created by a researcher, it was rated as “the item is appropriate (4)” and “the item is appropriate but minor changes can be made” by 5 experts, individually; they were languages experts and two were experts in health sciences. Content validity index (CVI) values were calculated using the Davis method, based on opinions such as “recommended (3)”, “the item is somewhat appropriate and major changes are required (2)” and “the item is not suitable (1)”. Accordingly, the items in the scale were given 4 and 3. CVI values were calculated by dividing the total number of experts who gave points to the total number of experts, and 0.800 was accepted as the criterion [47]. As a result of the analysis, the item CVI (I-CVI) value of the thirteenth, sixteenth, and twenty-sixth items was 0.800, and the I-CVI values of the other items were 1.000. To evaluate the overall content validity of the scale, the CVI (S-CVI) value of the scale was calculated by dividing the sum of the CVI values of each item by the number of items, and the content validity of the scale was obtained according to the calculated CVI values to test the content validity of the scale. It was determined to be good, and the final version of the scale was created.

### 2.3. Statistical Analysis

Data was analyzed by using SPSS 25.0 and the R package, version 4.0.2. First of all, expert statements for items were analyzed for the language and content validity of the Turkish version of the scale. The internal consistency of the scale was calculated with the Cronbach alpha value with a high reliability for the value of 0.70 or above [48]. Test–retest and internal consistency reliability analyses were used for reliability analysis. The relationship between the test–retest data was tested by Pearson correlation in order to determine the scale’s ability to give consistent results between applications and its invariance over time. The reliability level was determined by calculating the reliability coefficient of the final version of the scale. The content validity was analyzed by using the exploratory factor analysis (EFA) which was performed to determine the factor structure of the scale by removing items with factor loadings less than 0.40 or the difference between two factor loadings less than 0.10 from the scale. Confirmatory factor analysis (CFA) was performed to check the validity of the structure obtained by EFA, while the ratio of evaluated chi-square to degrees of freedom (χ^2^/df), root mean square error of approximation (RMSEA), comparative fit index (CFI), standardized root mean square residual (SRMR), Tucker–Lewis index (TLI), goodness of fit index (GFI), and incremental fit index (IFI) indexes were used to evaluate how well the data fits the model [49,50,51]. In addition, measurement invariance according to gender was examined to investigate whether the scale measured the same characteristics in different groups.

## 3. Results

According to the data obtained from the 312 participants who volunteered to the study and were evaluated as eligible for analysis, the frequency for females was found as 61.3%, whereas the frequency of males was 38.7%. Gender was chosen as the demographic variable to test measurement invariance in the Turkish adaptation of the PS-SGD scale.

### 3.1. Exploratory Factor Analysis

According to the results of EFA analysis, which was used to determine whether the adaptation of the PS-SGD scale to Turkish culture has the same structure as the original scale, the difference between the factor loadings of the 8th item (I apply my own methods to help me sleep) was found at 0.014, indicating that there was cross loading between the two dimensions. Since the loading between these two factors was less than 0.10, item 8 was removed from the scale [52]. Moreover, the factor loadings of the 18th item were found to be less than 0.40, indicating that item 18 could not be placed exactly in any of the dimension, and was therefore also removed from the scale. The factor loadings of the remaining items varied between 0.41 and 0.85. After the removal of both item 8 and item 18, the overall Kaiser–Meyer–Olkin (KMO) value of the final version of the Turkish version of the scale was calculated as 0.82 and Bartlett’s test of sphericity was found to be statistically significant (χ^2^ (66) = 2131.895, *p* < 0.0001), indicating that the data is suitable for principal component analysis (PCA). According to the results of the PCA, it was determined that there were six factors with eigenvalues exceeding one and the variance explained by these six factors for the scale was 56%.

Both the original and the Turkish version of the scale consists of six subscales, but some factor structures of the Turkish version appear to be different from the original scale. Since the factors are easy to interpret and the correlation between the sub-dimensions is low (r < 0.41), varimax rotation has been applied to the component loadings of the factors, which is presented in detail in Table 1. As a result of EFA analysis, in the Turkish version scale, unlike the original scale, items 1–5 were included in the “Emotional health (EH)” subscale, items 6 and 7 were included in a separate subscale called as “Social health (SH)”, while items 17 and 18, together with items 23–25, were combined and included in the “Health care and Physical activity (HC and PA)” subscale. The other items are in the same subscales as the original scale. Descriptive statistics for the scale and subscales and Cronbach alpha values for the subscales are given in Table 2.

### 3.2. Confirmatory Factor Analysis

**Figure 2:**
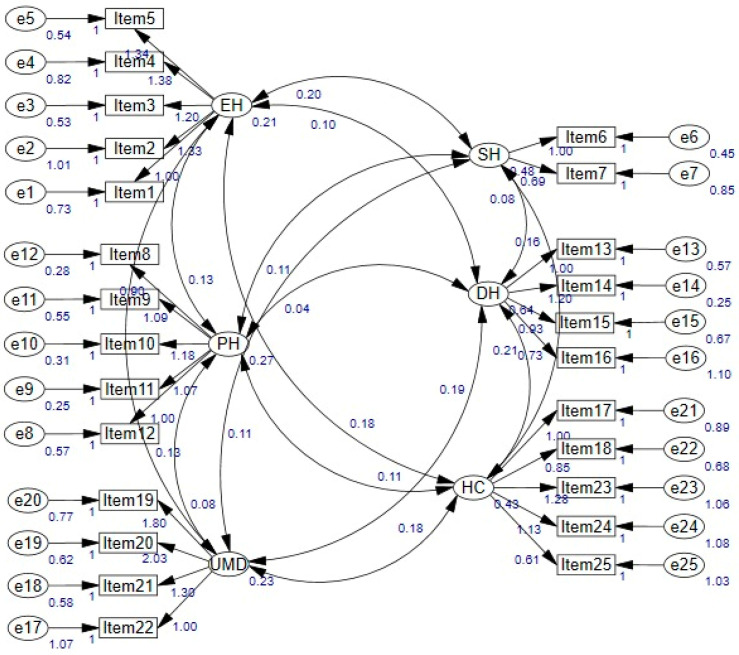
The CFA loading of PS-SGD.

To verify the six-dimensional structure obtained by EFA analysis for the adaptation of the PS-SGD scale to Turkish population CFA was conducted on 312 students, while Cronbach alpha value was obtained as 0.85 and the KMO value as 0.82. When items 8 and 18 were deleted from the scale, it was observed that the Cronbach’s alpha coefficient varied between 0.83 and 0.85. As a result, since a higher Cronbach’s alpha value was not achieved by removing items, it was determined that the Turkish version of the scale had a high reliability with 25 items. In addition, as a result of the split half method applied to the scale, the Alpa coefficient was calculated as 0.77 for the first half of the scale (13 items) and 0.79 for the second half (12 items), and the correlation between the two halves was obtained as 0.55. According to the split half method, reliability coefficients were evaluated with Spearman–Brown and Guttman Split-Half coefficients, and since the reliability coefficients were 0.71 and 0.70, respectively, it was concluded that the scale was highly reliable. Additionally, to test the invariance of the test results over time, PS-SGD was applied to 50 students with a 2-week interval, and the test–retest reliability of the scale was measured. The correlation coefficient for test–retest reliability obtained from 50 students who participated in both applications was calculated as r = 0.74, *p* < 0.001. As a result of these calculated findings, it was determined that the scale consisting of 25 items was reliable. CFA fit indices were calculated to check the validity of the five-factor structure obtained in EFA. According to the 25 items in the Turkish version of the scale, the fit indices were obtained as χ^2^/df (1.722), CFI (0.907), RMSEA (0.048), SRMR (0.057), and IFI (0.908). According to the findings presented in Table 3 and Figure 2, the fit indices were at acceptable fit values, thus confirming the acceptability and applicability of the adaptation of the PS-SGD scale to Turkish culture with a five-factor structure

The measurement invariance of the PS-SGD scale according to the gender as demographic variable was tested according to structural, metric, scale, and strict invariance hierarchy. ∆CFI and ∆RMSEA values were considered to determine whether measurement invariance was achieved between two hierarchical stages. In model comparisons, ∆CFI < 0.020 and ∆RMSEA < 0.030 thresholds were taken into account for structural and metric invariance, and ∆CFI < 0.010 and ∆RMSEA < 0.015 thresholds were taken into account for the metric model and scalar invariance model, as suggested by Rutkowski and Svetina [53]; in addition, as a second criterion for all metrics, a threshold of ∆CFI and ∆RMSEA < 0.01 among the hierarchical rankings of the models, recommended by Cheung and Rensvold [54], was also taken into account for the strict invariance model and other models.

According to the structural, metric, scale, and strict invariance findings for the PS-SGD scale presented in Table 4, fit values at good level in both gender groups indicate that all invariances are achieved (RMSEA < 0.08, SRMR < 0.08). In addition, the inter-metric ∆CFI and ∆RMSEA values were less than or equal to 0.01 and were in accordance with the threshold values recommended by Rutkowski and Svetina [53]. Accordingly, considering the goodness-of-fit statistics obtained from the analysis results conducted with the multi-group CFA method, it was seen that full measurement invariance was achieved according to gender. It can be interpreted that the item–factor structure and factor loadings, variances, covariances, and error variances of the PS-SGD scale are equivalent in male and female young adult groups.

## 4. Discussion

The aim of the study was to adapt the “Post-Pandemic Health Promotion Behaviour of Young Adults in the Digital Age” scale, originally developed by Heo et al. [47], to the Turkish population. The adaptation of the scale plays an important role in the assessment of health promotion behavior of the young Turkish population who have an online lifestyle and new health perception after the COVID-19 pandemic.

According to Pender [55,56], self-directed perception of human–environment interaction patterns is essential for behavioral change. In this regard, the Turkish-adapted scale can analyze, predict and verify the effectiveness of health promotion behavior in the new post-pandemic period, especially young adults who are digital natives using mobile devices and internet as a source for health information and all aspects of daily life [57].

In the present study, the PP-HPB was adapted to the Turkish population, however, the mostly used health promotion scale is HPLP-II, developed by Walker et al. [58,59]. While both scales have six factors, the sub-dimensions of HPLP-II are health responsibility, physical activity, nutrition, spiritual growth, interpersonal relationships, and stress management, whereas PP-HPB has combined rest and sleep together with self-management and interpersonal relationships under the psychosocial health sub-dimension in addition to the five other sub-dimensions termed as personal hygiene, dietary habits, health management, using mobile devices, and physical activity. According to the EFA and CFA analysis of the Turkish-adapted version of the scale, the item 8 related to sleep had to be removed due to its low factor load 0.014, while the psychosocial health factor was divided into two separate factors named as “emotional health” and “social health”. Thus, item 8 related with sleep could not be placed under one specific dimension. This might be due to the limited numbers of items related to sleep or sleep might be equally correlated to social and emotional health, as well as to the UMD sub-dimension, and could not be specifically combined to any of these sub-dimensions. Another reason might be misunderstanding of the item. In other words, the meaning of the phrase “I practice my own methods to help me sleep “might not be understood clearly.

In contrast to the original PP-HPB, items of health management and physical activity appeared under the same factor and were combined in the Turkish-adapted scale called as “health care and physical activity”. This was done by Woo [60], who suggests that any variables statistically highly correlated in an EFA can be grouped. Moreover, given its close relation to health, physical activity can be integrated into the health care sub-dimension rather than forming a separate one, since it is well accepted that physical activity is a key parameter in health care, especially in preventing and even treating non-communicable diseases. On the other hand, item 18 “I have balanced meals that include the three main nutrients; carbohydrates, proteins, and fats” was also removed from the scale according to EFA and CFA results, while this item also coded for the “Nutrition” dimension in HPLP-II created by Walker et al. [61] and used in the original PP-HPB. This dimension involves knowledgeable selection and consumption of foods essential for sustenance, health, and well-being, while it includes choosing a healthful daily diet consistent with guidelines provided by the food guide pyramid [62,63,64]. It has been widely accepted that the intake of each macronutrient must meet essential requirements while allowing for an adequate balance between protein, carbohydrates, and fats without exceeding calorie limits. A healthy dietary pattern is fundamental for health maintenance and disease prevention at all stages of life, while macronutrients are needed to support energy needs and meet physiologic requirements [65]. However, Turkey is a very large country, having a wide spectrum of diverse eating habits from the North to South and East to West regions. The nutritional habits are highly affected by cultural aspects in Turkey, which varies from region to region such that Mediterranean diet has been seen in the West and South of Turkey, while the eastern and southeastern Anatolia consumes mainly meat, and high carbohydrate consumption has been observed in the middle Anatolia region. Although item 18 plays an important role for healthy dietary pattern, it can be argued that most of the participants are not aware of the balance of macronutrients and lack the knowledge about intake recommendations of proteins, carbohydrates, and lipids. The dietary habits rely mostly on cultural food, without taking care of a balanced diet. This argument is supported by the findings of the study such that the lowest score of all sub-dimensions was the dietary habits (DH) sub-dimension, while the highest sub-dimension score was obtained in personal hygiene, as seen in Table 2. It is not surprising that the highest score after the pandemic was the “Personal Hygiene” since strict hygiene rules were applied everywhere, advertisements were made about hygiene rules and hand sanitizers were placed in shops, entrances to prevent the spread of the virus. Thus, during the pandemic and post-pandemic, personal hygiene became one of the most focused aspect of health promotion and it is reasonable that this sub-dimension scored the highest among other sub-dimensions.

In general, findings of the study were in accordance with the previous literature focused on healthy lifestyle behaviors in Turkish University students. Although Turkish University students from both medical [66] and dental [67] departments had learned about the health impacts of nutrition and physical exercise, even they scored low for these two sub-dimensions, which was explained by the environment of attending a university, which is characterized by a relatively stressful schedule that prevents students from physical activity participation and eating a healthy balanced diet. A study comparing the scores of medical and non-medical students concluded that, independent of the field of study, university students do not have healthy lifestyles in terms of diet and physical activity and have a tendency to be overweight or even obese [68].

On the other hand, dietary recommendations for carbohydrates and lipids are quite flexible [69], while the effects of foodstuffs go beyond the sum of individual nutrients and depend on the food matrix, which refers to the interaction of the physical structure and composition of food, involving both nutrient and non-nutrient components [70,71]. Similar to most of the world, the young Turkish generation consume processed food and fast food in the 21st century, effecting their dietary habits negatively, especially after the pandemic [72,73,74,75,76,77,78,79]. A systematic meta-analysis study reported an increase in disordered eating behavior, such as emotional eating, binge eating, and vomiting, and compulsive eating disorders associated with lower body image and increased weight during the COVID-19 pandemic period [80]. The COVID-19 pandemic period reduced psychological well-being [81], disturbed the emotional balance, and changed specific and general psychopathology of eating disorders [82].

As a result of EFA conducted to test the construct validity, the six-factor structure, each factor representing a sub-dimension, remained the same as the original scale, but the configuration or grouping of the items in the Turkish version differed from the original scale. In the final version of the scale, which has been adapted to Turkish society, the “EH” subscale contains items 1–5, “SH” subscale contains items 6–7, “PH” subscale contains items 8–12, “DH” subscale contains items 13–16, and “UMD” sub-dimension contains items 19–22. The sixth sub-dimension is combined and called “HC and PA” and contains items 17–18 in addition to items 23–25. The reliability of the Turkish version of the scale was found to be high, with a Cronbach alpha calculated at 0.85. Additionally, Cronbach’s alpha values of the sub-dimensions are 0.79 for the PH sub-dimension, 0.78 for the DH sub-dimension, 0.72 for the UMD sub-dimension, 0.70 for the EH sub-dimension, 0.68 for the HC and PA sub-dimensions, and 0.51 for the SH sub-dimension. Since the SH sub-dimension consists of two items, similar to the original scale, it is evident that the Cronbach alpha value is low. The six-subscale structure resulting from EFA was examined with CFA, and fit indices showed that the scale fit well with the six-factor structure. The results of multi-group CFA analysis, which tested the measurement invariance of the scale according to gender, showed that the post-pandemic health promotion behaviors of young adults between the ages of 19 and 29 years achieved structural, metric, scale, and strict measurement invariance, respectively. The means, variances, covariances, and error variances of the model in the male and female young adult groups were found to be equivalent in both groups.

Although the Turkish adaptation of the scale has been found as valid and reliable, some limitations of the study have to be addressed. One of the important limitations of the study was the sampling procedure. Despite the convenience sampling method, the PS-SGD was found to be a valid and reliable tool to determine post-pandemic health promotion behaviors for Turkish adults under the applied conditions. However, to generalize the results, the study should be repeated with a larger sample, including participants from different education levels and different economic levels living in different regions of Turkey. In our study, students were enrolled in one university only; thus, the results cannot be generalized to the total young population in the country. Furthermore, the study concerned only recreation students; therefore, its findings cannot be generalized to students in other fields. In future studies, the effects of different demographic variables such as ethnicity, family structures, health knowledge, and economic status on post-pandemic health promotion behaviors in young adults can be investigated and guiding results can be obtained for researchers. For data collection, a self-reported questionnaire was used; thus, participants’ responses may not reflect reality.

## 5. Conclusions

Findings of the study reveal that the Turkish-adapted version of the post-pandemic health promotion behaviors of young adults in the digital age (PS-SGD) scale is valid and reliable to assess the emotional and social health, personal hygiene, dietary habits, health management, use of mobile devices, and physical activity behavior of young adults in Turkish society. The adapted scale will serve as a reliable assessment tool for researchers, behavioral scientists, and health professionals who will focus on health promotion behavior in young adults in modern society in the post-pandemic period, considering the prevalence of digital lifestyles and unhealthy behaviors. The scale can be used to collect data to establish new health policies to change unhealthy behavior and also to measure the effectiveness of educational programs to improve health promotion behavior and decrease the risk of NCD. Further studies should be conducted in both similar and diverse settings at regular intervals to identify needs, use feasible interventions, and evaluate proceedings.

## Figures and Tables

**Table 1:** Factor loadings obtained by varimax rotation.

Items	Factor 1 (PH)	Factor 2 (DH)	Factor 3 (UMD)	Factor 4 (EH)	Factor 5 (HC and PA)	Factor 6 (SH)
Item 1				0.755		
Item 2				0.416		
Item 3				0.525		
Item 4				0.695		
Item 5				0.560		
Item 6						0.716
Item 7						0.744
Item 8	0.776					
Item 9	0.662					
Item 10	0.801					
Item 11	0.788					
Item 12	0.605					
Item 13		0.724				
Item 14		0.851				
Item 15		0.800				
Item 16		0.599				
Item 17					0.410	
Item 18					0.494	
Item 19			0.766			
Item 20			0.691			
Item 21			0.682			
Item 22			0.555			
Item 23					0.815	
Item 24					0.741	
Item 25					0.498	

**Table 2:** Descriptive statistics and reliability values for the scale and its sub-dimension.

Scale/Sub-Dimension	Mean	S.D.	Min.	Max.	Cronbach Alpha
PH	4.33	0.86	7	25	0.79
DH	2.79	1.13	4	20	0.78
UMD	3.11	1.16	4	20	0.72
EH	3.44	1.06	5	25	0.70
HC and PA	3.10	1.24	6	25	0.68
SH	3.65	1.03	2	10	0.51
Total	3.41	1.21	35	122	0.85

Min. and max. show the minimum and maximum total values that can be taken from the scale/sub-dimension, respectively.

**Table 3:** Fit indices for the models for the Turkish form of the PS-SGD in CFA.

	PS-SGD	Acceptable Fit	Perfect Fit
CMIN/DF	1.72	<5	<3
GFI	0.90	>0.90	>0.95
IFI	0.91	>0.90	>0.95
TLI	0.90	>0.90	>0.95
CFI	0.91	>0.90	>0.95
RMSEA	0.05	<0.08	<0.05
SRMR	0.06	<0.10	<0.05

**Table 4:** Fit statistics for measurement invariance according to gender.

	$\chi^{2}$	df	$\chi^{2}/df$	CFI	TLI	RMSEA	SRMR	∆CFI	∆RMSEA
Configural	776.115	520	1.492	0.877	0.858	0.056	0.066	-	-
Metric	811.225	539	1.505	0.869	0.854	0.057	0.071	−0.008	0.001
Scalar	872.211	558	1.563	0.849	0.837	0.060	0.076	−0.020	0.003
Strict	1085.93	605	1.794	0.834	0.829	0.062	0.078	−0.015	0.002

## Data Availability

The study data can be obtained by contacting the author of correspondence, Mustafa Can Koç, at cankoc_01@hotmail.com.
